# Engineered Zinc Finger Protein Targeting 2LTR Inhibits HIV Integration in Hematopoietic Stem and Progenitor Cell-Derived Macrophages: In Vitro Study

**DOI:** 10.3390/ijms23042331

**Published:** 2022-02-19

**Authors:** Koollawat Chupradit, Wannisa Khamaikawin, Supachai Sakkhachornphop, Chaniporn Puaninta, Bruce E. Torbett, Suparerk Borwornpinyo, Suradej Hongeng, Methichit Wattanapanitch, Chatchai Tayapiwatana

**Affiliations:** 1 Siriraj Center for Regenerative Medicine, Research Department, Faculty of Medicine Siriraj Hospital, Mahidol University, Bangkok 10700, Thailand; koollawat.chu@mahidol.ac.th (K.C.); methichit.wat@mahidol.ac.th (M.W.); 2 Center of Biomolecular Therapy and Diagnostic, Faculty of Associated Medical Sciences, Chiang Mai University, Chiang Mai 50200, Thailand; 3 Faculty of Medicine, King Mongkut’s Institute of Technology Ladkrabang, Bangkok 10520, Thailand; wannisa.kh@kmitl.ac.th; 4 Research Institute for Health Sciences, Chiang Mai University, Chiang Mai 50200, Thailand; ssakkhachornphop@yahoo.com; 5 Department of Anatomy, Faculty of Medicine, Chiang Mai University, Chiang Mai 50200, Thailand; chaniporn.puaninta@cmu.ac.th; 6 Department of Molecular and Experimental Medicine, The Scripps Research Institute, La Jolla, CA 92037, USA; betorbet@scripps.edu; 7 Department of Biotechnology, Faculty of Science, Mahidol University, Bangkok 10400, Thailand; bsuparerk@gmail.com; 8 Excellent Center for Drug Discovery, Mahidol University, Bangkok 10400, Thailand; suradej.hon@mahidol.ac.th; 9 Department of Pediatrics, Faculty of Medicine, Ramathibodi Hospital, Mahidol University, Bangkok 10400, Thailand; 10 Division of Clinical Immunology, Department of Medical Technology, Faculty of Associated Medical Sciences, Chiang Mai University, Chiang Mai 50200, Thailand; 11 Center of Innovative Immunodiagnostic Development, Faculty of Associated Medical Sciences, Chiang Mai University, Chiang Mai 50200, Thailand

**Keywords:** anti-HIV-1 scaffold, zinc finger protein, zinc finger domain, hematopoietic stem/progenitor cell, gene therapy

## Abstract

Human hematopoietic stem/progenitor cell (HSPC)-based gene therapy is a promising direction for curing HIV-1-infected individuals. The zinc finger protein (2LTRZFP) designed to target the 2-LTR-circle junction of HIV-1 cDNA was previously reported as an intracellular antiviral molecular scaffold that prevents HIV integration. Here, we elucidate the efficacy and safety of using 2LTRZFP in human CD34^+^ HSPCs. We transduced 2LTRZFP which has the mCherry tag (2LTRZFPmCherry) into human CD34^+^ HSPCs using a lentiviral vector. The 2LTRZFPmCherry-transduced HSPCs were subsequently differentiated into macrophages. The expression levels of pro-apoptotic proteins of the 2LTRZFPmCherry-transduced HSPCs showed no significant difference from those of the non-transduced control. Furthermore, the 2LTRZFPmCherry-transduced HSPCs were successfully differentiated into mature macrophages, which had normal phagocytic function. The cytokine secretion assay demonstrated that 2LTRZFPmCherry-transduced CD34^+^ derived macrophages promoted the polarization towards classically activated (M1) subtypes. More importantly, the 2LTRZFPmCherry transduced cells significantly exhibited resistance to HIV-1 integration in vitro. Our findings demonstrate that the 2LTRZFPmCherry-transduced macrophages were found to be functionally and phenotypically normal, with no adverse effects of the anti-HIV-1 scaffold. Our data suggest that the anti-HIV-1 integrase scaffold is a promising antiviral molecule that could be applied to human CD34^+^ HSPC-based gene therapy for AIDS patients.

## 1. Introduction

Antiretroviral therapy (ART) is the current standard treatment for HIV-1-infected patients. It is categorized into six different classes that affect different steps of HIV-1 replication [1]. In the past few decades, ART has been used as a single-tablet-a-day dose that contains a combination of the three most effective anti-viral drugs [2]. This combination can suppress HIV-1 replication to undetectable levels (≤50 copies/mL) and prevent HIV-1 infection of new cells; however, it cannot cure HIV [3]. ART does not eliminate latent HIV reservoirs in memory CD4^+^ T cells and macrophages [3,4]. Additionally, poor medication adherence or interruption of dosage can accelerate the emergence of HIV-1 drug resistance (HIVDR), which can lead to a viral rebound in peripheral blood [5]. To address this issue, several alternative approaches have been developed in the attempt to completely cure HIV. Despite the fact that several candidates are being tested in clinical trials, no effective HIV-1 vaccine has yet been developed [6]. In the meantime, alternative therapies, including broadly neutralizing antibodies [7], adoptive transfer of expanded cytotoxic T cells targeting latent HIV reservoirs [8], and chimeric antigen receptor T cells, are currently being investigated [9,10].

Given the limitations of ART and the unavailability of alternative therapies for HIV, hematopoietic stem/progenitor cell (HSPC) gene therapy is a promising approach to provide a long-term treatment of HIV-1-infected individuals with a one-time dose without a daily ART. Validation of the use of HSPC transplantation to cure HIV occurred in 2009 in the case of Timothy Ray Brown, known as “the Berlin patient,” who received an allogeneic stem cell transplant from a donor naturally immune to HIV-1 (CCR5Δ32 homozygous) [11]. More than ten years later, RNA detection indicated that he remains free of HIV-1 [12,13,14]. HSPCs have the ability to self-renew and differentiate into multilineage hematopoietic cell types including T lymphocytes and macrophages, which are the HIV-1 target cells. Therefore, HSPCs are an attractive tool for gene therapy to maintain life-long resistance to HIV infection or replication [15]. Transplantation of anti-HIV-1 gene-modified HSPCs in AIDS patients could continuously provide HIV-resistant hematopoietic cells throughout the life of a patient [16]. Hence, finding potential anti-HIV targets for use in combination with HSPC-based therapy could increase the chance of curing HIV.

Several anti-HIV-1 targets have been identified for use in HSPC-based therapy to achieve intracellular immunization, including RNA-based methods [17,18], C46 fusion inhibitor [19], revM10 dominant-negative protein [20], and gene editing proteins such as zinc finger nucleases (ZFNs), transcription activator-like effector nucleases (TALENs), and clustered regularly interspaced short palindromic repeats (CRISPR)/CRISPR-associated protein 9 (Cas9) [21,22]. However, these strategies have some restrictions. The extremely high mutation rate of the viral RNA genome, which allows evasion from the immune system, leads to insensitive binding of RNA-based molecules to their viral target sites [23,24]. Disruption of chemokine receptors such as CCR5 and CXCR4 by gene editing strategies can negatively affect the immune cell function and induce chronic inflammation [25,26]. Moreover, the unexpected adverse effects of CRISPR/Cas9 can occur due to the development of viral resistance to Cas9/sgRNA, resulting in replication-competent viral escape mutants [27]. Consequently, the discovery of anti-HIV-1 molecules that target more conserved regions of the HIV-1 genome and are not harmful to normal cell function could overcome these limitations.

2LTRZFP containing six adjacent ZFP motifs was explicitly designed to bind non-covalently to the 2LTR junction of HIV-1 DNA to interfere with the HIV-1 integration process [28]. The target of 2LTRZFP is a highly conserved region of HIV-1 and discrete to human cell compartments. It has been shown that the anti-HIV-1 gene activity significantly reduced HIV-1 integration and replication in human T cell lines and primary peripheral blood mononuclear cells (PBMCs) [28,29]. Moreover, the combination of 2LTRZFP with another anti-HIV-1 molecule, Ank^GAG^1D4, inhibited both HIV-1 integration and assembly [30]. Since the anti-HIV-1 activity of 2LTRZFP has not been evaluated in HSPCs, the present study aims to validate the efficiency of 2LTRZFP in HSPCs to retain the antiviral effects and induce their differentiation into mature macrophages, which are the early cellular targets of HIV-1 infection. Macrophages are more resistant to virally induced cytopathic effects of HIV-1 than CD4^+^ T lymphocytes, thus HIV-1-infected macrophages can produce viruses for more extended periods [31,32]. Functional analysis of 2LTRZFP in human HSPCs and human HSPC-derived macrophages will be an essential foundation for further study in animal models.

## 2. Results

### 2.1. Generation of Human CD34^+^ HSPCs Expressing 2LTRZFPmCherry

**Figure 1:**
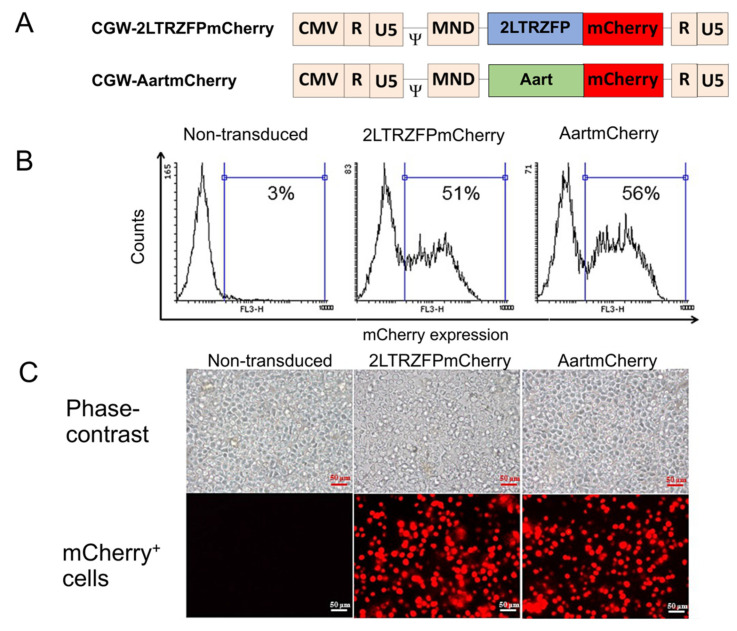
Transduction efficiency of the 2LTRZFPmCherry and AartmCherry lentiviral vectors in the CD34^+^ HSPCs; Ψ, packaging signal. (**A**) Schematic diagram of lentiviral vectors of CGW-2LTRZFPmCherry and CGW-AartmCherry (control). (**B**) Representative flow cytometric analysis of mCherry expression in the 2LTRZFPmCherry- and AartmCherry-transduced HSPCs. (**C**) Representative images of the transduced cells expressing mCherry under a fluorescence microscope. Scale bar = 50 µm.

HSPCs are frequently characterized by the surface expression of CD34 [33]. Downregulation of CD34 has been shown to correlate with loss of self-renewal and reflects the differentiated fate of HSPCs in cell culture. Here, CD34 was used as a marker to isolate HSPCs from G-CSF-mobilized peripheral blood samples from healthy donors using an anti-CD34 antibody conjugated to magnetic beads. To assess the safety of 2LTRZFP in human CD34^+^ HSPCs and validate the inhibition of HIV-1 integration, the CGW-2LTRZFPmCherry lentiviral vectors [30] were used for transduction into human CD34^+^ HSPCs. The isolated CD34^+^ HSPCs were cultured for three days before transduction with either CGW-2LTRZFPmCherry or CGW-AartmCherry control vectors at an MOI of 20 (Figure 1A). Aart is a six ZFP designed to recognize a unique 18 bp target sequence that is not present in the human genome [34]. Aart was used as a negative control for non-specific ZFP binding. Alignment of the amino acid sequences of 2LTRZFP and Aart shows 80% identity [28].

Flow cytometric analysis showed that we obtained 51% and 56% of the mCherry^+^ cells in human CD34^+^ HSPCs transduced with CGW-2LTRZFPmCherry and CGW-AartmCherry, respectively (Figure 1B). Fluorescence microscopy indicated that 2LTRZFPmCherry and AartmCherry were expressed in human CD34^+^ HSPCs (Figure 1C).

### 2.2. The 2LTRZFPmCherry-Transduced Cells Maintained Good Viability and Showed No Difference in Levels of Pro-Apoptotic Proteins after the Lentiviral Transduction

**Figure 2:**
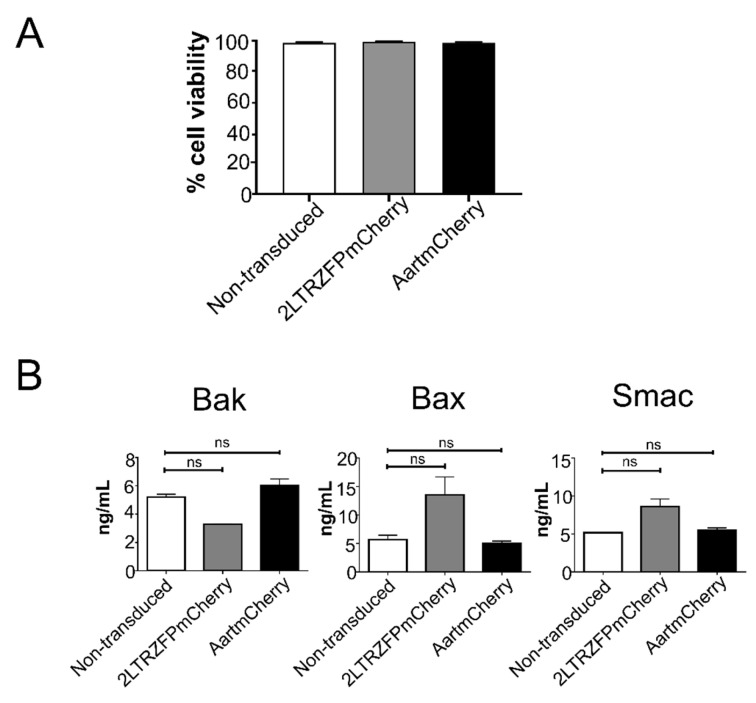
Viability of CD34^+^ HSPCs after transduction with the 2LTRZFPmCherry or the AartmCherry. (**A**) Percentages of cell viability of CD34^+^ HSPCs transduced with the 2LTRZFPmCherry (grey bar) or the AartmCherry (black bar), and the non-transduced cells (white bar). All data are represented as mean ± SEM. (**B**) The expression levels of pro-apoptotic proteins; Bak, Bax, and Smac in the CD34^+^ HSPCs transduced with 2LTRZFPmCherry or the AartmCherry compared to the non-transduced cells. Data are represented as mean ± SEM. (*n* = 3), *p* = 0.33, ns = not significant.

To determine whether the lentiviral gene transfer of 2LTRZFP affects cell viability in the transduced cells, the trypan blue dye exclusion method was performed in the transduced CD34^+^ HSPCs. We found that the viability of the transduced cells was not significantly different from the non-transduced CD34^+^ HSPCs (Figure 2A). In addition, the levels of pro-apoptotic proteins including Bak, Bax, and Smac in both 2LTRZFPmCherry- and AartmCherry-transduced cells were not significantly different from the non-transduced cells (*p* = 0.33, by Kolmogorov-Smirnov test) (Figure 2B). Taken together, these results demonstrated that anti-HIV-1 integrase 2LTRZFP did not affect cell viability or levels of pro-apoptotic proteins of the CD34^+^ HSPCs.

### 2.3. Functional Analyses of the CD34^+^ HSPC-Derived Macrophages Expressing 2LTRZFP

**Figure 3:**
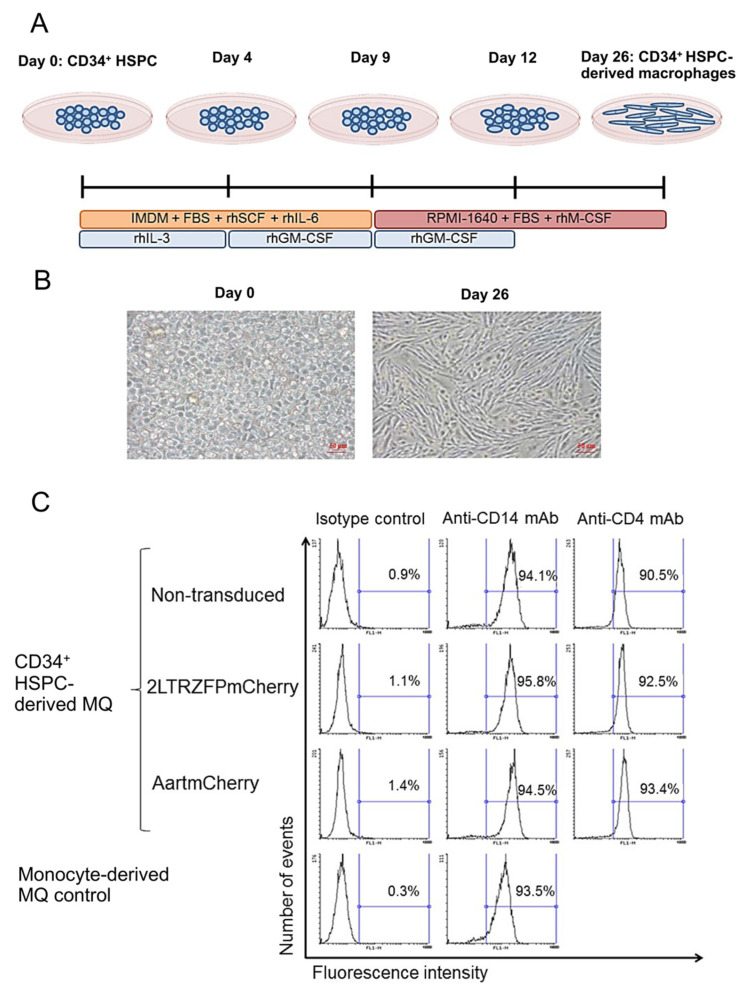
Differentiation of CD34^+^ HSPCs into macrophages. (**A**) The schematic timeline shows the differentiation of CD34^+^ HSPCs into macrophages. (**B**) The morphological changes of the CD34^+^ HSPCs in each stage of differentiation. (**C**) Representative flow cytometric analysis of CD14 and CD4 on the transgenic CD34^+^ HSPC-derived macrophages and the PBMC monocyte-derived macrophages. MQ = macrophage (s).

Macrophages play a critical role in the immune system, specifically in the inflammatory response. Since macrophages are typically distributed in tissues throughout the body, introducing anti-HIV genes into macrophages could prevent and eradicate latent HIV-1 reservoirs. We differentiated the 2LTRZFPmCherry- or AartmCherry-transduced cells to a myeloid lineage in cytokine culture media to generate mature macrophages (Figure 3A). Morphological analysis showed that the macrophages derived from CD34^+^ HSPCs had a elongated shape typical of macrophages (Figure 3B). We then investigated the levels of the monocyte/macrophage marker, CD14, and found that the 2LTRZFPmCherry transgenic macrophages expressed similar levels of CD14 when compared to those of the AartmCherry-transduced cells, the non-transduced cells, and the monocyte-derived macrophages that were purified from primary PBMCs. Similarly, the levels of CD4, a primary HIV-1 receptor on macrophages, and helper T lymphocytes in all samples were comparable (Figure 3C).

**Figure 4:**
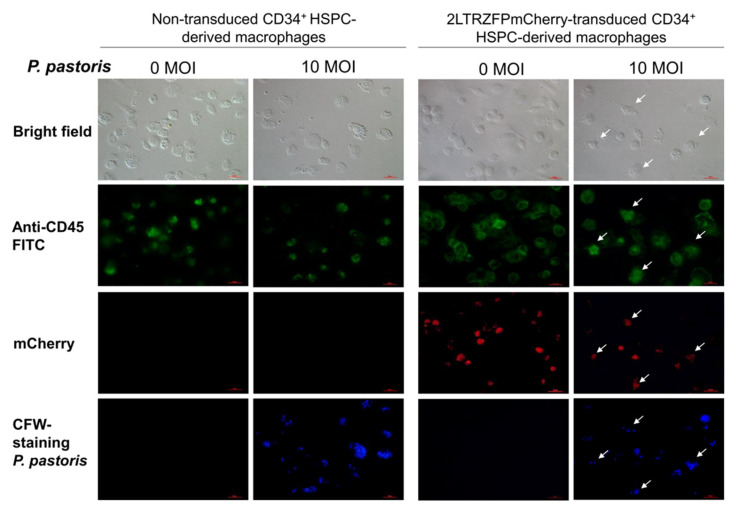
The phagocytic activity of the 2LTRZFPmCherry-transduced CD34^+^ HSPC-derived macrophages compared to the non-transduced CD34^+^ HSPC-derived macrophages. Green color (anti-CD45 FITC antibody) represents the CD34^+^ cell-derived macrophages. The 2LTRZFPmCherry and calcofluor-white (CFW) staining of *P. pastoris* were shown in red and blue colors, respectively (white arrows).

To determine the phagocytic function of the 2LTRZFPmCherry-transduced CD34^+^ HSPC-derived macrophages, we added the CFW-labelled *Pichia pastoris* at an MOI of 10 to the culture medium for 4 h. Phagocytosis was observed using fluorescent microscopy. The results showed that the 2LTRZFPmCherry transgenic CD34^+^ HSPC-derived macrophages had similar phagocytic activity as the non-transduced control (Figure 4).

### 2.4. Cytokine Secretion Assay of the CD34^+^ HSPC-Derived Macrophages Expressing 2LTRZFP

**Figure 5:**
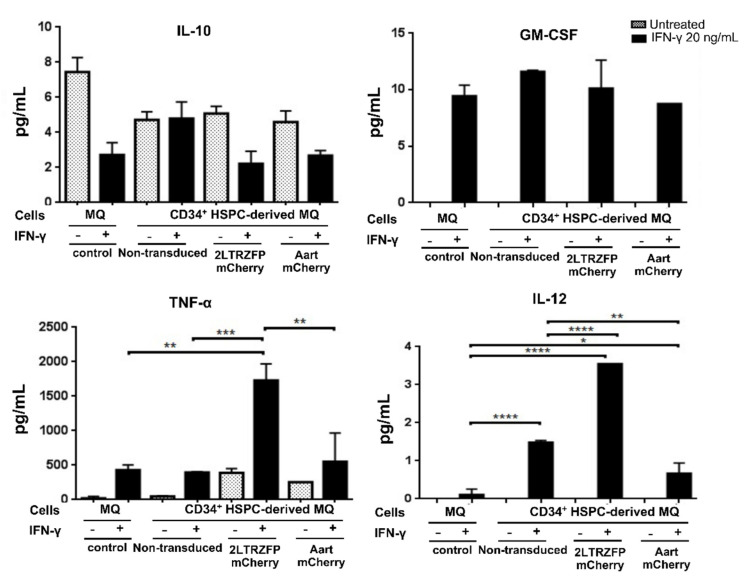
Cytokine secretion profiles of transgenic CD34^+^ derived macrophages. The non-transduced, AartmCherry, and 2LTRZFPmCherry transduced macrophages derived from CD34^+^ HSPCs, and PBMC monocyte-derived macrophages were stimulated with IFN-γ. At 24 h post-stimulation, supernatants were collected and assayed by cytokine bead array. Experiments were performed in duplicate. * *p* < 0.05, ** *p* < 0.01, *** *p* < 0.001, and **** *p* < 0.0001 from one-way ANOVA analysis. MQ = macrophages.

To determine whether the transgenic macrophages retained their functional capacity to secrete cytokines at similar levels to the primary monocyte-derived macrophages, the non-transduced, 2LTRZFPmCherry- or AartmCherry-transduced CD34^+^ HSPC-derived macrophages were stimulated with IFN-γ. The levels of secreted cytokines were measured by cytokine bead array. The results demonstrated no significant differences in levels of secreted IL-10 and GM-CSF among the non-transduced, 2LTRZFPmCherry-, AartmCherry CD34^+^ HSPC-derived macrophages, and the PBMC monocyte-derived macrophages. However, the levels of secreted IL-12 and TNF-α in the 2LTRZFPmCherry transduced macrophages were higher than those of the AartmCherry and the non-transduced control cells (Figure 5). The results indicated that the transgenic macrophages still provided the ability to produce the normal cytokine profiles similar to the non-transduced macrophages. Interestingly, the 2LTRZFPmCherry transduced macrophages displayed higher levels of IL-12 and TNF-α, suggesting that transduction of 2LTRZFPmCherry could increase the possibility of macrophages being polarized into the M1 (classically activated) subtype [35].

### 2.5. 2LTRZFP Mediated Inhibition of HIV-1 Integration in CD34^+^ HSPCs and Their Mature Macrophages

The success of anti-HIV gene therapy in HSPCs requires a safe and efficient gene delivery system, with inhibition of HIV-1 replication proficiency. The efficiency of 2LTRZFP to inhibit HIV-1 integration was evaluated in CD34^+^ HSPCs and CD34^+^ HSPC-derived macrophages. The non-transduced control, AartmCherry− and 2LTRZFPmCherry-transduced cells were infected with an X4-tropic HIV-1-based luciferase reporter virus pseudotyped with the envelope glycoprotein of vesicular stomatitis virus (VSV-G) (HIV-1_NL4-3.Luc.R−E−/VSV-G_) at 50 ng of p24 per 10^5^ cells. This non-replicative chimeric virus comprises the HIV-1 core (NL4-3), the luciferase reporter gene, and the VSV-G envelope glycoprotein. It enters all cells with equal efficiency, independent of CD4 expression levels, and integrates its genome to express luciferase as an indicator of HIV-1 infection and integration [36]. The levels of HIV-1 infection were evaluated at three days post-infection by detecting the luciferase activity.

**Figure 6:**
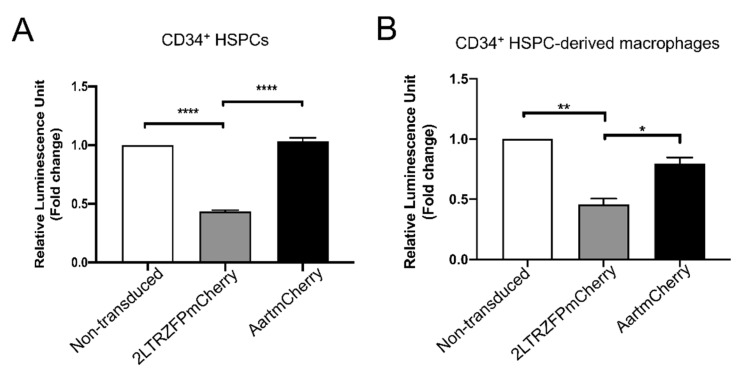
2LTRZFP-mediated inhibition of HIV-1 integration in the (**A**) transgenic CD34^+^ HSPCs and (**B**) transgenic macrophages. All data are represented as fold change of Relative Luminescence Unit (RLU) compared with the non-transduced control and presented as mean ± SEM. Experiments were performed in triplicate and duplicate, respectively. * *p* < 0.05, ** *p* < 0.01, and **** *p* < 0.0001 from one-way ANOVA analysis.

Both CD34^+^ HSPCs (Figure 6A) and CD34^+^ HSPC-derived macrophages (Figure 6B) expressing 2LTRZFPmCherry had significant protection against X4-tropic HIV-1 integration indicated by around 2.3- and 2.1-fold reduction of luciferase activity, respectively, when compared to the non-transduced control. As expected, CD34^+^ HSPCs and CD34^+^ HSPC-derived macrophages expressing AartmCherry did not have any anti-HIV effect. This result indicated that the lentiviral vectors efficiently delivered the 2LTRZFPmCherry genes and significantly inhibited the HIV-1 integration in the CD34^+^ HSPCs and their mature macrophages.

## 3. Discussion

HSPC-based gene therapy is considered a novel therapeutic approach to achieving long-term resistance against HIV infection and could potentially cure HIV/AIDS with a single treatment [4,15,16,37,38,39,40]. Gene therapy in human CD34^+^ HSPCs could prevent the infection of susceptible cells because HSPCs give rise to all blood cell types in HIV-1 pathogenesis, including CD4^+^ T cells, macrophages, dendritic cells, and microglia. These cells function as a source of HIV-resistant cells in central and mucosal lymphoid organs. Various gene-based therapies have been developed to inhibit different stages of HIV replication. The greatest challenge is to find safe and efficient anti-HIV gene therapy molecules for HSPC-based therapy. Specifically designed scaffold proteins are attractive for anti-HIV gene therapy due to their remarkable physical stability, simple expression in prokaryotic systems, and inexpensive production [41]. In addition, the binding sites of scaffold proteins are easily designed to specifically bind to target HIV-1 DNA, amino acid sequences, or crucial cell receptors to block HIV-1 entry into target cells.

This study focused on inhibiting HIV replication using anti-HIV-1 integrase molecular scaffolds, namely 2LTRZFP, a ZFP designed without *Fok*I endonuclease activity. The 2LTRZFP was designed to specifically target non-covalent 2-LTR circle junctions of HIV-1, which are highly conserved and essential sequences for HIV-1 integration without interfering with the host genome or proteome [28,29]. The benefit of the construct lacking *Fok*I nuclease is that it prevents off-target cleavage of the host genome. Here, we show that 2LTRZFP expression was achieved in human CD34^+^ HSPCs using the self-inactivating (SIN) CGW lentiviral vector transduction system, with at least 50% of mCherry^+^ cells at an MOI of 20. HIV-1 integration was significantly inhibited in human CD34^+^ HSPCs expressing 2LTRZFPmCherry, but not in those expressing the ZFP (AartmCherry) (negative control) and the non-transduced control.

Since 2LTRZFP is a new tool for HSPC-based gene therapy, it was necessary to evaluate its safety in HSPCs and their mature derivatives. We found that the introduction of the 2LTRZFPmcherry into human CD34^+^ HSPCs by the lentiviral vectors did not affect the cell viability and proliferation in the transduced cells. The transduced HSPCs could be differentiated into mature functional macrophages. There was no significant difference in the yields of mature macrophages upon cytokine induction. However, it is necessary to determine the distribution of the lentiviral vector common insertion sites to avoid benign integration bias and oncogenic selection [42]. Furthermore, as regenerative medicine advances, 2LTRZFP applied in induced pluripotent stem cells (iPSCs) could have the potential for generating a continuous supply of HIV-1-resistant HSPCs for transplantation into HIV-infected patients [43].

Using several strategies, stem cell-based HIV gene therapy is currently being tested in clinical trials. The history of major developments in HSPC-based gene therapy transplantation for AIDS patients from 1981 to date was reviewed by Hütter [4]. On-going and planned clinical trials of HSPC therapy for HIV-infected patients have been described by Kuritzkes [44]. Numerous approaches are currently under investigation, including allogeneic CCR5-deficient HSPC transplantation and autologous anti-HIV genetically modified HSPC transplantation. In early clinical trials, oncogenic gamma retroviruses, such as Moloney murine leukaemia virus (MMLV), were used to mediate gene transfer of the trans-dominant rev protein [45,46] or anti-HIV ribozyme-targeted *tat* and *vpr* genes [47] into CD34^+^ cells of HIV-positive patients. The results demonstrated the safety and feasibility of gene transfer and HSPC transplantation with no adverse effects. However, there was no significant long-term anti-HIV effect due to the low expression of the genes of interest and the production of a replication-competent virus, which are the primary potential hazards of this technology [48].

In a recent clinical study, retrovirus vectors were superseded by a lentiviral vector that enabled integration in the absence of cell division and prevented replication of the competent virus. The first clinical study that applied the lentiviral vector for anti-HIV gene delivery introduced a triple combination of a Tat/Rev shRNA, a TAR decoy, and a CCR5 ribozyme into CD34^+^ cells of patients with AIDS-related non-Hodgkin lymphoma (NHL). However, the outcome showed low levels of anti-HIV gene markers in 0.2–0.32% PBMCs at up to 24 months after reinfusion [49].

In addition to the stability of anti-HIV modified cells and cell transplantation protocol, an essential requirement of a successful HIV-1 gene therapy applied in HSPCs is to maintain phenotypically and functionally normal differentiated cells and restore their immunological function. Accordingly, the 2LTRZFP-transduced CD34^+^ HSPCs were differentiated to mature macrophages. We demonstrated that the 2LTRZFP transgenic macrophages expressed normal levels of CD14 and CD4 surface markers as compared to those of the non-transduced, AartmCherry-transduced, or PBMC monocyte-derived macrophages. This result indicated that the transgenic macrophages were phenotypically normal.

To further investigate the function of macrophages, we stimulated the 2LTRZFPmCherry-transduced macrophages with IFN-γ to induce polarization into the M1 (classically activated) subtype. The M1 subtype expressed IL-12^high^, IL-23^high^, IL-10^low^ and secreted inflammatory cytokines including IL-1β, TNF, and IL-6, which promote inflammation and Th1 polarization of CD4 cells [50,51]. These results indicated that introducing the 2LTRZFPmCherry gene in CD34^+^ cell-derived macrophages had no apparent effect on IL-10 and GM-CSF secretions compared to the AartmCherry transgenic and non-transduced cells [52,53]. Interestingly, the levels of TNF-α and IL-12 were up-regulated in the 2LTRZFPmCherry-transduced macrophages suggesting that the 2LTRZFPmCherry could affect the polarization of macrophages towards the classically activated M1 state. However, further study on this effect should be performed in an animal model, which provides a better physiological environment than the in vitro system.

Our previous work demonstrated that 2LTRZFP inhibited the viral integration process in SupT1 cells [29,30,54]. In this study, we use the X4-tropic HIV-1 pseudotyped luciferase reporter virus since the virus needs to integrate into the host chromosome and then trigger luciferase expression. Thus, the protection of 2LTRZFP against HIV integration can be determined. The human CD34^+^ HSPCs and CD34^+^ HSPC-derived macrophages expressing 2LTRZFP revealed moderate protection against HIV integration, as evidenced by the X4-tropic HIV-1 pseudotyped luciferase reporter assay. Further analysis of 2LTRZFP anti-HIV-1 activity in mouse models is required to determine their suitability and safety before use in the human field [55,56]. The NOD *scid* gamma (NSG) and humanized bone marrow/liver/thymus (BLT) mouse models are small animal models that permit evaluation of anti-HIV-1 gene therapy of human CD34^+^ HSPCs. The reconstitution and differentiation into mature human leukocytes and HIV-1 infection can be assessed in these models to determine anti-HIV-1 gene-modified HSPC properties in vivo [39,56,57,58,59,60,61]. Experiments in non-human primate models are necessary to further characterize this molecular scaffold’s biosafety and antiviral activity in immune systems more closely related to that of humans.

## 4. Materials and Methods

### 4.1. Ethical Approval and Consent to Participate

The human CD34^+^ HSPCs from individual donors were acquired using protocols approved by the Committee on Human Rights Related to Research Involving Human Subjects, Faculty of Medicine, Ramathibodi Hospital, Mahidol University (study approval code: ID 06-56-07). Written informed consent was obtained from each individual. All methods were carried out in accordance with relevant guidelines and regulations.

### 4.2. Isolation and Culture of Human CD34^+^ HSPCs

Human CD34^+^ HSPCs were isolated from granulocyte-colony-stimulating factor (G-CSF)-mobilized peripheral blood using the CD34 MicroBead Kit (Miltenyi Biotec, Auburn, CA, USA) [62]. The purity of human CD34^+^ HSPCs was determined by co-staining with anti-human CD34 mAbs (BioLegend, San Diego, CA, USA) and anti-human CD45 mAbs (BioLegend) and analyzed by flow cytometry. Purified human CD34^+^ HSPCs were frozen in a cryopreservation medium containing 10% dimethyl sulfoxide (DMSO; Sigma-Aldrich, St. Louis, MO, USA) and 90% Fetal Bovine Serum (FBS; Thermo Scientific HyClone, Cramlington, UK). Human CD34^+^ HSPCs were thawed and cultured in Iscove’s Modified Dulbecco’s Medium (IMDM; Gibco, Grand Island, NY, USA) containing 100 ng/mL recombinant human stem cell factor (rhSCF), 50 ng/mL recombinant human interleukin-3 (rhIL-3), and 50 ng/mL recombinant human interleukin-6 (rhIL-6) (all from Cell Guidance Systems, Cambridge, UK), and 10% FBS. Cultures were maintained in a 37 °C humidified incubator containing 5% CO_2_.

### 4.3. Generation of Human CD34^+^ HSPCs Stably Expressing 2LTRZFPmCherry or CGW-AartmCherry by Lentiviral Gene Transfer

Frozen human CD34^+^ HSPCs were thawed and cultured in IMDM containing 10% FBS, 100 ng/mL rhSCF, 50 ng/mL rhIL-3, and 50 ng/mL rhIL-6 for three days before transduction with either the CGW-2LTRZFPmCherry lentiviral vector or the CGW-AartmCherry control lentiviral vector at an MOI of 20. After adding the virus, the cells were subjected to spinoculation by centrifugation at 2000× *g* at 32 °C for 1.5 h, in the medium containing 4 µg/mL of Polybrene (Sigma–Aldrich, St. Louis, MO, USA). The cells were then transferred to a humidified incubator and maintained at 37 °C and 5% CO_2_ for 24 h. The next day, the transduced cells were washed three times with a fresh medium and further cultured in the fresh cytokine medium. The transduction efficiency was determined at day three post-transduction by fluorescence microscopy and flow cytometry (FACSCalibur™; BD Biosciences, Le Pont-de-Claix, France).

### 4.4. Determination of Cell Viability by Trypan Blue Exclusion Assay

Aliquots of non-transduced, 2LTRZFPmCherry- and AartmCherry-transduced CD34^+^ HSPCs were centrifuged at 500× *g* for 5 min. The cell pellets were then resuspended in a proper amount of PBS and subsequently stained with 0.4% Trypan Blue solution (Gibco). The unstained (viable) and stained (nonviable) cells were counted using a hemacytometer and the percentages of viable cells were determined.

### 4.5. Apoptosis Assay

Total protein was extracted from 3 × 10^6^ HSPCs. According to the manufacturer’s instruction, the apoptosis assay was performed using the Bio-Plex Pro™RBM Apoptosis Assays (Bio-Rad Laboratories, Hercules, CA, USA). Then, 30 µL of 250 µg/mL of each protein sample was used to assess pro-apoptotic proteins (Bak, Bax, and Smac) using the Bio-Plex Multiplex System (Bio-Rad Laboratories, Hercules, CA, USA).

### 4.6. Differentiation of the 2LTRZFPmCherry-Transduced HSPCs into Mature Macrophages

The HSPCs were differentiated into macrophages, which are the HIV-1 target cells, following the previously published protocol [63]. Briefly, cells were cultured in a complete medium: IMDM containing 10% FBS, 100 U/mL penicillin (Gibco), 100 μg/mL streptomycin (Gibco), and 2 mM L-glutamine (Gibco), supplemented with 100 ng/mL rhSCF, 50 ng/mL rhIL-3, and 50 ng/mL rhIL-6 for four days. After four days, the medium was replaced with a complete medium supplemented with 50 ng/mL rhSCF, 50 ng/mL rhIL-6, and 50 ng/mL recombinant human GM-CSF (rhGM-CSF; Cell Guidance Systems) for five days. The cells were then cultured in the macrophage medium: RPMI-1640 medium (Gibco) containing 10% FBS, 100 U/mL penicillin, 100 μg/mL streptomycin, and 2 mM L-glutamine, supplemented with 50 ng/mL rhGM-CSF and 100 ng/mL recombinant human macrophage colony-stimulating factor (rhM-CSF; Cell Guidance Systems) for three days. The medium was replaced with the macrophage medium supplemented with 100 ng/mL rhM-CSF for 14 days. The expression of mCherry was examined by fluorescence microscopy. Macrophage surface markers were assessed by staining with anti-human CD14 mAbs (BioLegend) and CD4 mAbs (clone MT4/3; generously provided by Prof. Watchara Kasinrerk, Chiang Mai University, Chiang Mai, Thailand), and analyzed by flow cytometry (FACSCalibur™; BD Biosciences, Franklin Lakes, NJ, USA).

### 4.7. Measurement of Phagocytosis of Human CD34^+^ HSPC-Derived Macrophages

HSPC-derived macrophages expressing 2LTRZFPmCherry were seeded at a density of 2 × 10^5^ cells/well of a 24-well plate and maintained in RPMI-1640 medium containing 100 ng/mL rhM-CSF at 37 °C and 5% CO_2_ for 14 days. The mCherry expression was examined by fluorescence microscopy. Next, the cell wall of *P. pastoris* was stained with calcofluor-white (CFW). The differentiated macrophages were co-cultured with the CFW-labelled *P. pastoris* at an MOI of 10 and maintained at 37 °C and 5% CO_2_ for 4 h. After 4 h, the co-cultured cells were washed three times with 1X PBS, and the cell membrane of macrophages was labelled with anti-human CD45-FITC (ImmunoTools GmbH, Friesoythe, Germany). The phagocytosis was determined by confocal microscopy.

### 4.8. Assessment of Cytokine Production in CD34^+^ HSPC-Derived Macrophages

CD34^+^ HSPC-derived macrophages expressing either 2LTRZFP or AartmChery were seeded at a density of 1 × 10^5^ cells/well of a 24-well plate and maintained at 37 °C and 5% CO_2_ for 24 h. The cells were stimulated with 20 ng/mL of interferon-gamma (IFN-γ) (R&D Systems, Minneapolis, MN, USA) for 24 h in a humidified incubator at 37 °C and 5% CO_2_. The cell-free culture supernatant was collected and stored at −80 °C for measuring cytokine production using Bio-plex Pro^TM^ Human Cytokine Th1/Th2 panel (Bio-Rad, Hercules, CA, USA). The non-transduced CD34^+^ cell-derived macrophages and unstimulated cells were used as controls.

### 4.9. VSV-G-Pseudotyped HIV-1_NL4-3.Luc.R_^−^_.E_^−^ Infection

Human CD34^+^ HSPCs and their derived mature macrophages stably expressing 2LTRZFPmCherry or AartmCherry were tested for antiviral integration by challenging with VSV-G-pseudotyped HIV-1_NL4-3.Luc. R_^−^_.E_^−^ . First, cells were seeded at a density of 1 × 10^5^ cells/well of a 96-well plate in 150 µL RPMI-1640 medium containing 100 ng/mL rhM-CSF for 24 h. The cells were treated with 50 µL VSV-G-pseudotyped HIV-1_NL4-3.Luc. R_^−^_.E_^−^ (50 ng of HIV-1 p24 per 10^5^ cells) and centrifuged at 2000× *g* at 32 °C for 1.5 h. The cells were then transferred to a humidified incubator and maintained at 37 °C and 5% CO_2_ for 72 h. The infected cells were examined for viral integration by measuring luciferase expression (Steady-Glo^®^ Luciferase Assay System; Promega Corporation, Madison, WI, USA). The luminescent signal was detected using the BioTek Synergy™ 4 Hybrid Microplate Reader (BioTek Instruments, Winooski, VT, USA).

### 4.10. Statistics

Statistical analyses were performed using GraphPad Prism software. Statistical significance was determined by Kolmogorov-Smirnov test and one-way ANOVA analysis. We indicated other significance levels as follows: *p* < 0.05 *, *p* < 0.01 **, *p* < 0.001 ***, *p* < 0.0001 ****.

## Data Availability

Any data or material that support the findings of this study can be made available by the corresponding author upon request.
